# Different Resting-State Functional Connectivity Alterations in Smokers and Nonsmokers with Internet Gaming Addiction

**DOI:** 10.1155/2014/825787

**Published:** 2014-11-18

**Authors:** Xue Chen, Yao Wang, Yan Zhou, Yawen Sun, Weina Ding, Zhiguo Zhuang, Jianrong Xu, Yasong Du

**Affiliations:** ^1^Department of Radiology, Ren Ji Hospital, School of Medicine, Shanghai Jiao Tong University, Shanghai 200127, China; ^2^Department of Child & Adolescent Psychiatry, Shanghai Mental Health Center, Shanghai Jiao Tong University, Shanghai 200030, China

## Abstract

This study investigated changes in resting-state functional connectivity (rsFC) of posterior cingulate cortex (PCC) in smokers and nonsmokers with Internet gaming addiction (IGA). Twenty-nine smokers with IGA, 22 nonsmokers with IGA, and 30 healthy controls (HC group) underwent a resting-state fMRI scan. PCC connectivity was determined in all subjects by investigating synchronized low-frequency fMRI signal fluctuations using a temporal correlation method. Compared with the nonsmokers with IGA, the smokers with IGA exhibited decreased rsFC with PCC in the right rectus gyrus. Left middle frontal gyrus exhibited increased rsFC. The PCC connectivity with the right rectus gyrus was found to be negatively correlated with the CIAS scores in the smokers with IGA before correction. Our results suggested that smokers with IGA had functional changes in brain areas related to motivation and executive function compared with the nonsmokers with IGA.

## 1. Introduction

The Internet is one of the most important media for communication and social interaction in modern life. However, a loss of control over Internet use results in disturbing negative consequences [1], such as obsession with gaming, lack of real-life relationships, lack of attention, aggression and hostility, stress, and decreased academic achievement [2–4]. This behavioral phenomenon has been named Internet addiction (IA) [1], or “Internet use disorder.” IA consists of at least three subtypes: Internet gaming addiction (IGA), sexual preoccupations, and email/text messaging [5]. In China, the most important subtype of IA is IGA [6]. Clinical evidence suggests that individuals with IA experience a number of biopsychosocial symptoms and consequences, such as salience, mood modification, tolerance, withdrawal symptoms, conflict, and relapse, which were traditionally associated with substance-related addictions, although it does not cause the same type of physical problems as other addictions such as alcohol or drug abuse [7, 8]. It was reported that the prevalence of IA was 10.7 percent in youth in China [9]. Because the number of Internet users is increasing rapidly, IA has become a serious public health problem.

Studies concerning various factors related to IA are conducted actively to understand and solve Internet addiction phenomenon. In light of behavioral addiction, researchers have been making efforts to find an association between IA and other problem behaviors which can lead to addiction, such as alcohol drinking and drug abuse [10]. Several studies have reported that the risk of IA is associated with an increased prevalence of substance dependence [11–13]. Sung et al. reported that the risk of IA was associated with cigarette smoking, alcohol drinking, drug abuse, and sexual intercourse among Korean adolescents [10]. Ko et al. [14] reported that Taiwanese adolescents with IA were more likely to have experience with substance use, including tobacco, alcohol, or illicit drugs. Ko et al., found that students addicted to the Internet and students experienced with substance use shared common personality characteristics more vulnerable to addiction. Similar findings among Greek adolescents were reported by Fisoun et al. [15]. These studies suggested that adolescents at high risk of IA may have personalities vulnerable to any addiction; these personalities have increased risk for substance use and sexual intercourse, which can lead to addiction. The overlap between IA and substance abuse and dependence may be due to similar characteristics predisposing toward and brain regions responding to Internet or substance use [11]. Individuals with IA and substance addiction share similar temperaments. Furthermore, similar functional alterations of brain regions such as dorsolateral and orbitofrontal cortices were found in subjects with IGA, drug addiction, and pathological gambling [16, 17]. Sung et al. proposed that it should not be interpreted that IA causes other problem behaviors among adolescents; however, it is likely that the same causal factors responsible for IA increase the risk of IA in adolescents engaging in other problem behaviors. Therefore, it appeared reasonable to consider concurrent problem behaviors, especially smoking, drinking, drug abuse, and sexual intercourse, when dealing with adolescents with a high risk of IA [10]. But, thus far, the brain functional changes in subjects with IA with and without substance addiction remain unclear. In our previous research, we found altered rsFC with PCC in IGA [18]. Therefore, in the present study, we aimed to determine whether subjects with IGA and substance addiction showed greater changes in rsFC compared with those with IGA without substance addiction.

The last decade has witnessed an explosion in the number of functional connectivity (FC) studies using fMRI, largely because FC allows for the exploration of large scale networks and their interactions, thus moving towards a systems-level understanding of brain functioning [19, 20]. This emerging neuroimaging tool has provided researchers with additional insights and spurred novel theories about the underlying neural substrates of various neuropsychiatric disorders [21]. In the present study, we compared resting-state functional connectivity (rsFC) with PCC between smokers and nonsmokers with IGA and a healthy control group. The aims of this study were (1) to detect the differences in rsFC with PCC alteration in smokers and nonsmokers with IGA and (2) to determine whether there were any relationships between altered rsFC with PCC and the severity of IGA and nicotine dependence.

## 2. Materials and Methods

### 2.1. Participants

Twenty-nine smokers with IGA, 22 nonsmokers with IGA, and 30 healthy controls (HC group) participated in the present study. The IGA groups were recruited from the Outpatient Department of Shanghai Mental Health Center. The control group was recruited through advertisements. All participants in the smoking group began smoking 2-3 years before study onset. Nicotine-dependent subjects are particularly suited as a comparison group for IGA because the neurotoxic effects of nicotine are limited compared with those of other drugs, such as alcohol [22, 23].

A basic questionnaire was used to collect demographic information such as gender, age, and final year of schooling completed. This study was approved by the Ethics Committee of Ren Ji Hospital, School of Medicine, Shanghai Jiao Tong University. The participants and their parents or legal guardians were informed of the aims of our study before the magnetic resonance imaging (MRI) examinations were conducted. Full and written informed consent was obtained from the parents or legal guardians of each participant.

All subjects were screened for psychiatric disorders with the Mini International Neuropsychiatric Interview (MINI) [24]. The recruitment criteria were age of 16–23 years, male gender, and being right-handed. A detailed explanation of the study was given, and, subsequently, informed consent was obtained from all participants. All subjects were interviewed by a psychiatrist to confirm the diagnoses of IGA and nicotine dependence. The criteria for IGA were assessed according to the modified Diagnostic Questionnaire for Internet Addiction (i.e., the YDQ) criteria by Beard and Wolf [25], and the criteria for nicotine dependence were assessed using the appropriate questions from the Structured Clinical Interview for DSM-IV [26]. None of the participants in the control groups had ever smoked.

The exclusion criteria included a history of any of the following: substance use disorders other than nicotine addiction, previous hospitalization for psychiatric disorders or a history of major psychiatric disorders, neurological illness or injury, mental retardation, and intolerance of magnetic resonance imaging.

### 2.2. Clinical Assessments

Five questionnaires were used to assess the participants' clinical features, namely, the Chen Internet Addiction Scale (CIAS) [27], Self-Rating Anxiety Scale (SAS) [28], Self-Rating Depression Scale (SDS) [29], Barratt Impulsiveness Scale-11 (BIS-11) [30], and the Fagerstrom Test of Nicotine Dependence (FTND) [31]. The CIAS, developed by Chen, contains 26 items on a 4-point Likert scale; it represents the severity of Internet addiction. The FTND is a six-item self-report questionnaire [31]. Scores can range from 0 (nondependent) to 10 (highly dependent). All questionnaires were initially written in English and then translated into Chinese.

### 2.3. MRI Acquisition

MRI was conducted using a 3T MRI scanner (GE Signa HDxt 3T, USA). A standard head coil with foam padding was used. During resting-state fMRI, the subjects were instructed to keep their eyes closed, remain motionless, stay awake, and keep the mind clear of any specific subjects. A gradient-echo echo-planar sequence was used for functional imaging. Thirty-four transverse slices (repetition time (TR) = 2000 ms, echo time (TE) = 30 ms, field of view (FOV) = 230 × 230 mm, 3.6 × 3.6 × 4 mm voxel size) aligned along the anterior commissure-posterior commissure line were acquired. Each fMRI scan lasted 440 s. Several other sequences were also acquired, including (1) 3D Fast Spoiled Gradient Recalled sequence (3D-FSPGR) images (TR = 6.1 ms, TE = 2.8 ms, TI = 450 ms, slice thickness = 1 mm, gap = 0, flip angle = 15°, FOV = 256 mm × 256 mm, number of slices = 166, 1 ×1 × 1 mm voxel size). (2) axial T1-weighted fast field echo sequences (TR = 331 ms, TE = 4.6 ms, FOV = 256 × 256 mm, 34 slices, 0.5 × 0.5 × 4 mm voxel size), and (3) axial T2W turbo spin-echo sequences (TR = 3013 ms, TE = 80 ms, FOV = 256 × 256 mm, 34 slices, 0.5 × 0.5 × 4 mm voxel size). The smokers with IGA did not smoke prior to scanning.

### 2.4. Statistical Analysis

For group comparisons of demographic and clinical measures, one-way ANOVA tests were performed using SPSS 18 (Statistical Package for the Social Sciences) to examine differences in the three groups, and Bonferroni post hoc tests were performed to examine differences between each pair of groups. A two-tailed *P* value of 0.05 was considered statistically significant for all analyses.

Structural brain MRI scans (T1- and T2-weighted images) were inspected by two experienced neuroradiologists. No gross abnormalities were observed in either group. Functional MRI preprocessing was performed using the Data Processing Assistant for Resting-State fMRI (DPARSF V2.3) (Yan & Zang, 2010, http://www.restfmri.net) which is based on Statistical Parametric Mapping software (SPM8) (http://www.fil.ion.ucl.ac.uk/spm) and the Resting-State fMRI Data Analysis Toolkit (REST, http://www.restfmri.net) [32, 33].

Data from each fMRI scan contained 220 time points. The first 10 volumes of each functional time-series were discarded because of the instability of the initial MRI signal and the initial adaptation of participants to the situation, and the remaining 210 images were preprocessed. The images were subsequently corrected for slice timing and realigned to the first image by rigid-body head movement correction (patient data exhibiting movement greater than 1 mm with maximum translation in* x*,* y*, or* z*, or 1° maximum rotation about the three axes, were discarded). No participant was excluded because of movement. The functional images were normalized into standard stereotaxic anatomical Montreal Neurological Institute (MNI) space. The normalized volumes were resampled to a voxel size of 3 mm × 3 mm × 3 mm. The echo-planar images were spatially smoothed using an isotropic Gaussian filter of 4 mm full width at half maximum.

The time-series in each voxel was detrended to correct for linear drift over time. Eight nuisance covariates (time-series predictors for white matter, cerebrospinal fluid, and the six movement parameters) were sequentially regressed from the time-series. Subsequently, temporal filtering (0.01–0.08 Hz) was applied to the time-series of each voxel to reduce the impact of low-frequency drift and high-frequency noise [34–37].

Posterior cingulate cortex (PCC) has attracted much research attention recently [38]. As a central component of the proposed DMN, the PCC is implicated in attentional processes. Previous studies have demonstrated that PCC neurons respond to reward receipt, magnitude, and visual-spatial orientation [39, 40]. Our previous research also revealed that IGA subjects had lower gray matter density in the left posterior cingulate cortex, and connectivity with the PCC was positively correlated with CIAS scores in the right PCC [18, 41]. Additionally, Dong et al. found that IGA subjects showed higher fractional anisotropy (FA), indicating greater white matter integrity, in the left PCC relative to healthy controls [42]. Thus, PCC was used in the present study as the ROI seed. The PCC template, which consisted of Brodmann's areas 29, 30, 23, and 31, was selected as the region of interest (ROI) using WFU-Pick Atlas software [43]. The blood oxygenation level-dependent signal time-series in the voxels within the seed region were averaged to generate the reference time-series. For each subject and seed region, a correlation map was produced by computing the correlation coefficients between the reference time-series and the time-series from all other brain voxels. Correlation coefficients were then converted to *z* values using Fisher's* z*-transform to improve the normality of the distribution [36]. The individual* z*-scores were entered into SPM8 for a one-sample* t*-test to determine the brain regions with significant connectivity to the PCC within each group. Individual scores were also entered into SPM8 for random effect analysis and one-way ANOVA tests were performed. Multiple comparison correction was performed using the AlphaSim program in the Analysis of Functional Neuroimages software package, as determined by Monte Carlo simulations. Statistical maps of the two-sample* t*-test were created using a combined threshold of *P* < 0.05 and a minimum cluster size of 54 voxels, yielding a corrected threshold of *P* < 0.05. Then, further group interaction analyses were performed with two-sample* t*-tests to identify the regions exhibiting significant differences in connectivity to the PCC between two groups based on the result of ANOVA analysis by using the result of the *F*-test as a mask to limit the *t*-tests to the significant regions. Multiple comparison correction was performed using the AlphaSim program. Regions exhibiting statistically significant differences were masked on MNI brain templates.

We also examined the relationship between CIAS scores and* z*FC in smokers and nonsmokers with IGA group. First, each cluster that demonstrated between-group differences in a group comparison of smokers with IGA versus nonsmokers with IGA was saved as a ROI. Then, the* z*FC values of each ROI were extracted by the REST software. Finally, the correlation analysis with* z*FC value of each ROI with CIAS and FTND in smokers with IGA was performed. A two-tailed *P* value of 0.00625 with Bonferroni correction was considered statistically significant.

## 3. Results and Discussion

### 3.1. Demographic and Clinical Results

Table 1 lists the demographic and clinical measures for each group. There were no significant differences in the distributions of age and years of education in the three groups. The smokers with IGA had higher CIAS (*P* < 0.001), SAS (*P* = 0.002), SDS (*P* < 0.001), and BIS-11 scores (*P* < 0.001) than healthy controls. The nonsmokers with IGA had higher CIAS (*P* < 0.001) and BIS-11 scores (*P* < 0.001) than healthy controls. No differences were found between IGA subgroups on clinical assessments.

### 3.2. Analysis of PCC Connectivity

#### 3.2.1. Three-Group ANOVA Analysis

**Figure 1:**
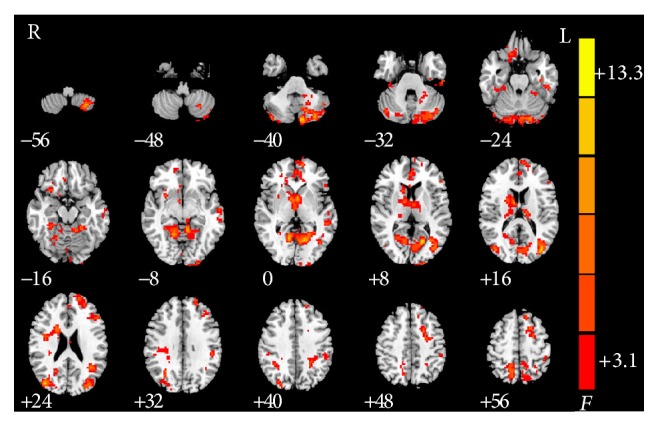
Significant between-group differences in rsFC of different brain regions with PCC between smokers with IGA, nonsmokers with IGA, and HC subjects. Note: the left part of the figure (L) represents the participant's left side, (R) represents the participant's right side. rsFC: resting-state functional connectivity; HC: healthy control; PCC: posterior cingulate cortex.

Significant difference of rsFC with PCC was found in left side of cerebellum posterior lobe, calcarine cortex, inferior temporal gyrus, middle temporal gyrus, middle occipital gyrus, inferior frontal gyrus, medial prefrontal gyrus, angular gyrus, inferior parietal lobule, superior frontal gyrus, precuneus, and superior frontal gyrus, as well as right side of rectus gyrus, insula, caudate, middle occipital gyrus, postcentral gyrus, and superior parietal lobule (Table 2 and Figure 1).

#### 3.2.2. Between-Group Analysis of PCC Connectivity: Smokers with IGA* versus* HC Group

**Figure 2:**
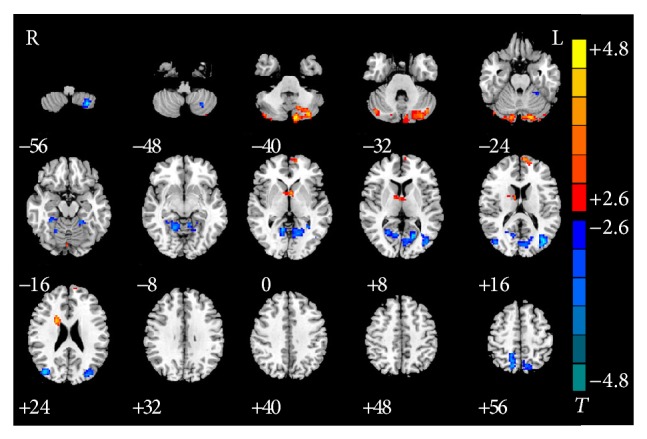
Significant between-group differences in rsFC of different brain regions with PCC between smokers with IGA and HC subjects. Compared with the HC group, the smokers with IGA exhibited increased rsFC in the bilateral cerebellum posterior lobe, bilateral caudate, and left medial frontal cortex. And decreased rsFC were found in the bilateral middle temporal gyrus, bilateral superior parietal lobules, left posterior cerebellum lobe, and right lingual gyrus (*P* < 0.05, AlphaSim-corrected). The *t*-score bars are shown on the right. Red indicates smokers with IGA > HC and blue indicates smokers with IGA < HC. Note: the left part of the figure (L) represents the participant's left side; (R) represents the participant's right side. rsFC: resting-state functional connectivity; HC: healthy control; PCC: posterior cingulate cortex.

Compared with the HC group, the smokers with IGA exhibited increased rsFC in the bilateral posterior cerebellar lobes, bilateral caudate, and left medial frontal cortex. In addition, decreased rsFC was found in the bilateral middle temporal gyrus, bilateral superior parietal lobules, left posterior cerebellum lobe, and right lingual gyrus (Table 3 and Figure 2).

#### 3.2.3. Between-Group Analysis of PCC Connectivity: Nonsmokers with IGA* versus* HC Group

**Figure 3:**
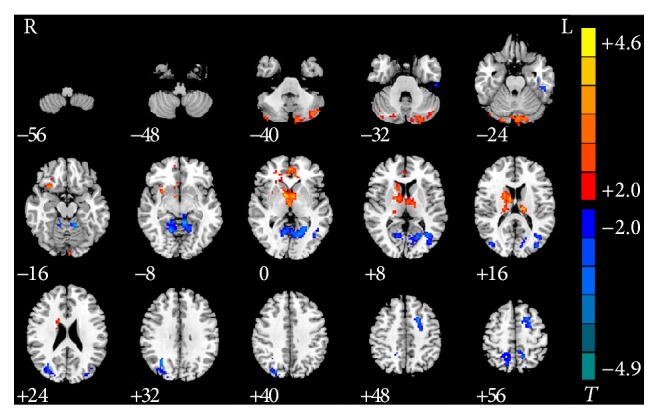
Significant between-group differences in rsFC of different brain regions with PCC between nonsmokers with IGA and HC subjects. Compared with the HC group, nonsmokers with IGA exhibited increased rsFC in left cerebellum posterior lobe, left medial prefrontal cortex, right caudate, and right insula. Decreased rsFC was found in left calcarine cortex, right superior parietal lobule, right middle occipital gyrus, left middle frontal gyrus, left precuneus, and left inferior temporal gyrus (*P* < 0.05, AlphaSim-corrected). The *t*-score bars are shown on the right. Red indicates nonsmokers with IGA > HC and blue indicates nonsmokers with IGA < HC. Note: the left part of the figure (L) represents the participant's left side; (R) represents the participant's right side. IGA: Internet gaming addiction; rsFC: resting-state functional connectivity; HC: healthy control, PCC: posterior cingulate cortex.

Nonsmokers with IGA exhibited increased rsFC in left cerebellum posterior lobe, left medial prefrontal cortex, right caudate, and right insula, compared with the HC group. Decreased rsFC was found in left calcarine cortex, right superior parietal lobule, right middle occipital gyrus, left middle frontal gyrus, left precuneus, and left inferior temporal gyrus (Table 5 and Figure 3).

#### 3.2.4. Between-Group Analysis of PCC Connectivity: Smokers with IGA* versus* Nonsmokers with IGA

**Figure 4:**
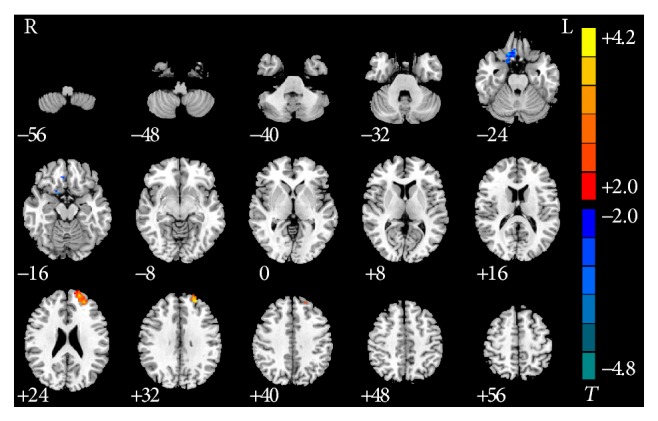
Significant between-group differences in rsFC of middle frontal gyrus and right rectus gyrus with PCC between smokers and nonsmokers with IGA. Compared with nonsmokers with IGA, the smokers with IGA exhibited increased rsFC in the left middle frontal gyrus and decreased rsFC in the right rectus gyrus (*P* < 0.05, AlphaSim-corrected). The *t*-score bars are shown on the right. Red indicates smokers with IGA > nonsmokers with IGA and blue indicates smokers with IGA < nonsmokers with IGA. Note: the left part of the figure (L) represents the participant's left side; (R) represents the participant's right side. IGA: Internet gaming addiction; rsFC: resting-state functional connectivity; PCC: posterior cingulate cortex.

Compared with nonsmokers with IGA, the smokers with IGA exhibited increased rsFC in the left middle frontal gyrus and decreased rsFC in the right rectus gyrus (Table 4 and Figure 4).

### 3.3. Correlation between PCC Connectivity and the Severity of IGA and Nicotine Dependence in the Smokers with IGA Group

The* z*FC values of the right rectus gyrus with PCC correlated with the CIAS (*r* = −0.476, *P* = 0.009) and FTND (*r* = −0.125, *P* = 0.52) in the smokers with IGA. No significant correlation was found in the* z*FC values of right middle frontal gyrus with the CIAS or FTND score. No significant correlation survived after Bonferroni correction.

### 3.4. Discussion

Numerous functional imaging studies have detected the possible neural mechanisms of the IGA and suggested that it may share psychological and neurobiological abnormalities with addictive disorders with and without substance abuse [6, 18, 44–46]. In agreement with the results of our previous study on IGA [18], similar areas with rsFC with PCC changes were found in smokers and nonsmokers with IGA compared with the control group in the current study, such as the cerebellum posterior lobe, caudate, medial frontal cortex, superior parietal lobules, insula, and precuneus. This finding implied that IGA individuals with/without substance addiction share some similar functional brain alterations. These brain areas were reported in the previous studies of cravings in IGA. The caudate nucleus contributes to stimulus-response habit learning, where behavior becomes automatic and hence is no longer driven by action-outcome relationships [47]. The insula and medial frontal lobes are consistently activated in imaging studies of craving [48, 49]. It was also suggested that the cerebellum is essential in craving induced by IGA, especially during preparation, execution, working memory [50], and fine-motor processes modulated by extrapyramidal systems.

The point we would like to emphasize in this study is that we compared rsFC with PCC in the subjects with IGA with/without nicotine dependence and found that the smokers with IGA exhibited increased rsFC in the left middle frontal gyrus and decreased rsFC in the right rectus gyrus. Furthermore, the PCC connectivity with the right rectus gyrus was negatively correlated with the CIAS scores in the smokers with IGA before correction, which suggested that the strength of the rsFC between PCC and right rectus gyrus may represent the severity of IGA in this group, and right rectus gyrus may play an important role in the pathogenesis of behavior combined substance addiction. The rectus gyrus is part of the orbitofrontal cortex (OFC), and the OFC is involved in the evaluation of reward of stimuli and the explicit representation of reward expectancy for substances [44], so the recuts gyrus has consistently been implicated in the pathology of both drug and behavioral addictions. Hong et al., [50] confirmed that male adolescents with Internet addiction have significantly decreased cortical thickness in the right lateral OFC. The extensive connections of the OFC with the striatum and limbic system suggest that it integrates emotion and natural drive from limbic and subcortical areas to assess the reward value against previous experience [51]. The OFC creates and maintains expectations of possible reward related to reinforcement [52]. Dorsolateral prefrontal cortex (DLPFC) is well known to be involved in working memory [53]. It is connected with other cortical areas and serves to link the present sensory experience to memory of past experiences in order to direct and generate appropriate goal-directed action [45, 46]. Thus, when substance cues are present and a positive expectancy has been generated, the DLPFC may contribute to maintaining and coordinating representations received from other regions during the craving response [52]. Our research found that, compared with the nonsmokers with IGA, the smokers with IGA showed decreased rsFC with PCC in rectus gyrus, suggesting they had abnormal function in OFC, which may lead to subjects having strong expectations of games or nicotine, and increased rsFC in DLPFC, supposing they had deficits in controlling appropriate behavior.

Despite the findings about IGA and behavior combined substance addiction, there are several limitations associated with this study which we would like to discuss. Firstly, this study focused on the Internet gaming subgroup of IA, but no direct comparisons were made with other IA subgroups; therefore it remains to be investigated how well the results may be extrapolated to other IA subgroups, if at all. Secondly, subjects with comorbid major psychiatric disorders or substance use disorders, other than nicotine, were excluded in this study. Thus, there is a limitation in generalizing the results of subjects of online gaming addiction to other substance using disorders and major psychiatric disorders. Thirdly, the present study was cross sectional, and we did not have information on the order of the onset of IGA and nicotine dependence. Thus, rsFC with PCC abnormalities in the smokers and nonsmokers with IGA may represent preexisting vulnerabilities or changes resulting from IGA or nicotine dependence behaviors/symptoms. Fourth, a smoker-only group shall be included in future studies for completeness. Fifth, the correlation results did not last when we adopted multiple comparisons (Bonferroni correction), which means that this should only be considered as an exploratory analysis. To increase the statistical power, the findings should be repeated with a larger sample of subjects. Finally, because participants in the present study were all young males, future work is needed to determine if the findings may be extended to other gender and age groups.

## 4. Conclusion

In summary, rsFC with PCC provides a useful tool for studying multifaceted neuropsychiatric diseases such as addiction at systems-level of assessment. Our results suggest that IGA individuals with/without substance addiction share some similar functional alterations in brain areas related to craving. IGA with substance addiction showed functional changes in areas involved in motivation, such as frontal rectus gyrus, and executive systems, such as the dorsolateral prefrontal cortex, compared with the IGA without substance addiction. These two areas may be candidate markers for identifying IGA individuals with and without substance addiction and should be investigated in future studies.

## Figures and Tables

**Table 1:** Demographic and personality characteristics of the three groups.

	Smokers with IGA (*n* = 29)	Nonsmoker with IGA (*n* = 22)	HCs (*n* = 30)	*F* value (*P* value)	*P*1-2	*P*1-3	*P*2-3
	(Mean ± SD)	(Mean ± SD)	(Mean ± SD)				
Age (years)	22.14 ± 2.54	21 ± 2.33	20.80 ± 2.91	2.08 (0.132)			
Education (years)	10.17 ± 1.91	11.00 ± 1.37	10.5 ± 2.18	1.251 (0.29)			
Chen Internet Addiction Scale (CIAS)	78.69 ± 7.61	74.55 ± 8.98	41.60 ± 9.15	157.59 (<0.001)	0.08	<0.001	<0.001
Self-Rating Anxiety Scale (SAS)	56.38 ± 12.54	49.09 ± 9.24	46.37 ± 10.42	6.49 (0.002)	0.063	0.002	0.99
Self-Rating Depression Scale (SDS)	58.07 ± 9.70	52.36 ± 9.93	47.76 ± 8.42	9.26 (<0.001)	0.094	<0.001	0.24
Barratt Impulsiveness Scale-11 (BIS-11)	63.41 ± 9.36	61.41 ± 8.43	47.77 ± 6.81	28.62 (<0.001)	0.9	<0.001	<0.001
FTND	6.51 ± 2.11						

SD: standard deviation; HC: healthy controls; IGA: Internet gaming addiction; FTND: Fagerstrom Test of Nicotine Dependence.

*P*1-2 for smokers with IGA group versus nonsmokers with IGA group, *P*1-3 for smokers with IGA group versus HC group.

*P*2-3 for nonsmokers with IGA group versus HC group.

**Table 2:** Summary of functional connectivity changes in three groups.

	Peak MNI coordinate region	Peak MNI coordinates			Number of cluster voxels	Peak *F* value
		*x*	*y*	*z*		
1	Left cerebellum, posterior lobe	−30	−60	−45	90	10.53
		−33	−81	−36	744	12.18
2	Left calcarine cortex (BA17)	−18	−66	6	1372	13.28
3	Left inferior temporal gyrus (BA20)	−48	−27	−27	80	6.93
4	Left middle temporal gyrus (BA21)	−63	−15	−12	88	7.73
5	Left middle occipital gyrus (AB17)	−18	−103	6	85	8.46
6	Left inferior frontal gyrus (BA45)	−48	27	21	109	6.90
7	Left medial prefrontal gyrus (BA10)	−9	63	18	222	7.56
8	Left angular gyrus (BA39)	−39	−54	24	58	8.79
9	Left inferior parietal lobule (BA40)	−53	−32	45	71	5.37
10	Left superior frontal gyrus (BA6)	−18	9	60	191	7.45
11	Left precuneus (BA5)	−12	−57	60	251	7.14
12	Left superior frontal gyrus (BA8)	−12	27	54	77	6.26
13	Right rectal gyrus (BA11)	6	24	−21	69	7.11
14	Right insula (BA48)	30	18	−15	59	6.46
15	Right caudate	14	−1	15	746	10.03
16	Right middle occipital gyrus (BA39)	42	−81	21	258	10.62
17	Right postcentral gyrus (BA3)	39	−26	36	74	5.89
18	Right superior parietal lobule (BA5)	15	−60	60	276	9.93

MNI: Montreal Neurological Institute; IGA: Internet gaming addiction; BA: Brodmann's area.

(*P* < 0.05, AlphaSim-corrected.)

**Table 3:** Summary of functional connectivity changes in smokers with IGA compared with the HC group.

	Peak MNI coordinate region	Peak MNI coordinates			Number of cluster voxels	Peak *t* value
		*x*	*y*	*z*		
1	Left middle temporal gyrus (BA39)	−39	−75	15	532	−4.64
2	Left cerebellum, posterior lobe	−30	−60	−51	60	−4.61
3	Right superior parietal lobule (BA7)	15	−69	63	186	−4.61
4	Left superior parietal lobule (BA7)	−15	−66	66	89	−4.41
5	Right middle temporal gyrus (BA39)	42	−81	21	105	−4.29
6	Right lingual gyrus (BA19)	15	−54	−6	228	−4.28
7	Left caudate	−3	6	0	60	4.01
8	Left medial prefrontal cortex (BA10)	−9	63	18	64	4.13
9	Right cerebellum posterior lobe	45	−72	−30	79	4.27
10	Right caudate	21	9	21	57	4.27
11	Left cerebellum posterior lobe	−33	−81	−36	421	4.77

MNI: Montreal Neurological Institute; HC: healthy control; IGA: Internet gaming addiction; BA: Brodmann's area.

Note: *t* > 0 indicates smokers with IGA > HC group in functional connectivity; *t* < 0 indicates smokers with IGA < HC group in functional connectivity.

(*P* < 0.05, AlphaSim-corrected.)

**Table 5:** Summary of functional connectivity changes in smokers with IGA compared with nonsmokers with IGA.

	Peak MNI coordinate region	Peak MNI coordinates			Number of cluster voxels	Peak *t* value
		*x*	*y*	*z*		
1	Right frontal rectus gyrus (BA11)	6	21	−21	67	−3.47
2	Left dorsolateral prefrontal cortex (BA9)	−24	51	33	120	3.92

MNI: Montreal Neurological Institute; IGA: Internet gaming addiction; BA: Brodmann's area.

Note: *t* > 0 indicates smokers with IGA > nonsmokers with IGA in functional connectivity,

*t* < 0 indicates smokers with IGA < nonsmokers with IGA in functional connectivity.

(*P* < 0.05, AlphaSim-corrected.)

**Table 4:** Summary of functional connectivity changes in nonsmokers with IGA compared with the HC group.

	Peak MNI coordinate region	Peak MNI coordinates			Number of cluster voxels	Peak *t* value
		*x*	*y*	*z*		
1	Left calcarine cortex (BA19)	−24	−60	6	711	−4.99
2	Right superior parietal lobule (BA7)	24	−63	63	186	−3.93
3	Right middle occipital gyrus (BA19)	30	−66	33	161	−3.90
4	Left middle frontal gyrus (BA8)	−21	6	48	143	−3.72
5	Left precuneus (BA5)	−12	−57	60	65	−3.69
6	Left inferior temporal gyrus (BA20)	−48	−27	−27	63	−3.63
7	Right insula (BA48)	27	21	−18	55	3.43
8	Left medial prefrontal cortex (BA10)	−3	48	3	57	3.51
9	Right caudate	6	0	−6	489	4.14
10	Left cerebellum posterior lobe	−21	−84	−27	384	4.58

MNI: Montreal Neurological Institute; IGA: Internet gaming addiction; HC: healthy control; BA: Brodmann's area.

Note: *t* > 0 indicates smokers with IGA > HC group in functional connectivity; *t* < 0 indicates smokers with IGA group < HC group in functional connectivity.

(*P* < 0.05, AlphaSim-corrected.)
